# Knowledge, attitudes and perceptions regarding malaria: a cross-sectional study in pregnant women attending antenatal care in the New-Bell district hospital, Douala, Cameroon

**DOI:** 10.11604/pamj.2020.36.207.16180

**Published:** 2020-07-22

**Authors:** Corine Blondo Kangmo Sielinou, Damian Anong, Samuel Nambile Cumber, Rosaline Yumumkah Cumber, Theresa Nkuo-Akenji

**Affiliations:** 1 Department of Microbiology and Parasitology, Faculty of Science, University of Buea, Buea, Cameroon,; 2 Department of Microbiology and Parasitology, Faculty of Science, University of Bamenda, Bamenda, Cameroon,; 3 Centre for Health Systems Research & Development, University of the Free State, Bloemfontein, South Africa,; 4 Office of the Dean, Faculty of Health Sciences, University of the Free State, Bloemfontein, South Africa,; 5 School of Health Systems and Public Health, Faculty of Health Sciences, University of Pretoria, Pretoria, South Africa,; 6 Institute of Health and Care Sciences, Sahlgrenska Academy, University of Gothenburg, Gothenburg, Sweden,; 7 Department of Political Science, University of KwaZulu-Natal, Durban, South Africa

**Keywords:** Pregnant women, malaria, Cameroon

## Abstract

**Introduction:**

malaria remains a major public health problem in Cameroon. For a successful malaria control, there is a need to evaluate the level of awareness, attitude and perception of people living in malaria endemic areas such as the swampy littoral region of Cameroon.

**Methods:**

this was a descriptive cross-sectional study targeting pregnant women attending ANC in the New-Bell District Hospital. Data was collected with a semi-structured questionnaire on demographic characteristics as well as knowledge, attitudes and practices regarding malaria.

**Results:**

two hundred and six pregnant women were enrolled in the study, all of them had heard on malaria in the past, with hospitals and television been the most known information dissemination channels. Only 60.2% of them own and used a LLINs with only 51.6% of owners treating the net.

**Conclusion:**

respondents with no education had poor knowledge on malaria. There is a need to improve education on malaria with active participation of women and improve malaria surveillance that will lead to malaria eradication.

## Introduction

Malaria is ancient but, it still remains a major public health problem in Cameroon and the major cause of death worldwide [1]. Despite being preventable and treatable, malaria continues to have a devastating impact on people´s health and livelihood around the world [2]. Globally, approximately about 216 millions of cases of malaria occur annually [3]. Approximately 445,000 deaths were attributed to malaria in 2016 with 91% of deaths recorded in sub-Saharan Africa [3]. In Cameroon, about 2 millions of cases were registered by the national surveillance system with up to 4000 deaths attributed to malaria yearly [4]. In malarious endemic area, pregnant women are at high risk of contracting the disease [5] with severe outcomes compared to their non-pregnant peers [6]. Malaria is a major cause of death in newborns and infants under 5 years, pregnant women, immuno-suppressed individuals, travelers and migrants from malaria non-endemic zones. The severity of malaria in newborn is due to the fact that, acquired immunity from mother against the pathogen is waning [7]. Malaria in pregnant women is more complicated and can lead to severe outcomes such as anemia, abortion, anemia, low birth weight, neonatal death. Preventing malaria is of great importance in ameliorating the quality of life, reducing the morbidity and mortality in a country [8]. Antenatal care (ANC) is the set of medical procedures and care carried out during pregnancy. They aimed at maintain pregnant women´s health and prevent neonatal death [9]. In Cameroon, only about 85% of pregnant women attend at least one ANC before delivery [10].

Knowing that malaria diagnosis, treatment and prevention protect women and baby´s health, there is a need to encourage pregnant women to attend ANC and improve education on malaria prevention and treatment. The third Sustainable Development Goals (SDGs) and the global technical strategy for malaria set visions to accelerate progress to eliminate malaria but, Cameroon is still amongst the most affected countries with transmission occurring throughout the year. Malaria is the main cause of consultation and hospitalization in health facilities [11] and is a disease linked to poverty [12]. The human and economic costs associated with declining quality of life, consultations, treatments, hospitalizations and other events related to malaria are enormous and often lead to low productivity and lost incomes [13]. Hence, prevention and proper treatment of malaria is important to improve the quality of life of people in malaria endemic zones. Malaria prevention and control activities are developed by the National Malaria Control Program (NMCP) of Cameroon. This mainly focuses on vector control by distributing long lasting insecticide treated mosquito nets; chemoprevention with the free distribution of intermittent preventive treatment against malaria to pregnant women, distribution of prophylaxis to prevent seasonal malaria in risky population in the northen regions and free treatment of malaria in children under 5 years all through the country [11]. The aim of the present study was to explore knowledge, attitudes and perceptions regarding malaria of pregnant women attending ANC in the New-Bell district hospital.

## Methods

A cross-sectional descriptive study was undertaken to assess the KAP regarding malaria among pregnant women attending ANC in the New-Bell district hospital from July to November 2014. Data were collected through interviews using a semi-structured questionnaire. The New-bell district hospital was selected for this study because, it is at the 4^th^category of public hospitals according to the Cameroon health system, it is easily accessible, delivers several services and has a high quality technical platform. Inclusion criteria for participants were consenting women with a confirmed pregnancy and ageing between 15 and 49 years. Pregnant women who did not consent to participate or with proven mental disturbances, or aged below 15 or above 49 years were excluded from the study. For the purpose of sample size estimation n, confidence level was set as 95% (Z=1.96), variance in the population P set at 30% and absolute error of margin sets as 0.5% (d=0.05). The minimum sample size estimated to be used was 384 pregnant women. However, due to limited time for the study, a finite population correction was done using:

$$\text{n=}\frac{{\text{z}}^{2}\text{P}\left(1-\text{P}\right)}{{\text{d}}^{2}}$$

n= (1.96)^2^x0.3(1-0.3)/[(0.05)]^2^

n = 322.7, sample size for an infinite population, n_0_=323 pregnant women. However, due to the limited time and inadequate time for the study, a finite population correction was done using:

$$\text{n}=\frac{{\text{n}}_{0}}{\left(1+{\text{n}}_{0}/\text{N}\right)}$$

n= 323/(1+323/2250)

n=282. Where: n=sample size for the finite population of pregnant women to be surveyed; n_0_=assumed sample size for an infinite population of pregnant women; N=Number of pregnant women attending ANC in a district hospital* which is 2250 in this context. According to the Cameroon reorientation of primary health care to the national health development plan [14], a district hospital is constructed for 45000 inhabitants with pregnant representing 5% of the populations. Sample size for the finite population for the study, n=282 pregnant women to be surveyed. A total of 282 pregnant women were enrolled in the study but, only 206 completely participated in the study, given a response rate of 73.0%.

**Data collection and analysis**: data were collected using a developed and pre-tested questionnaire with close-ended and opened questions. Directives on question answering were given to participants and written on the forms. For women who could not write or could not speak neither of the 2 official languages (English and French), a trained personnel was allocated to help in answering. Data collected were keyed in Microsoft Excel work sheet, cleared, transferred to Epi Info version 3.5.3. Statistical analyses done here were all descriptive in nature estimating the proportion of key indicators.

**Ethical consideration:** ethical clearance N° 2014/241/UB/FHS/IRB was obtained from the University of Buea Faculty of Health Science Institutional Review Board. Administrative authorizations N° 1206/L/MINSANTE/DRSPL/CSSE and N° 74/NS/MISANTE/DRSPL/DSNB/SSDNB obtained from the Littoral Regional Delegation of Public Health and the New-Bell District Health Service and permission for research exercise was granted by the head of the New-Bell district hospital. All study participants were granted verbal consent before questionnaire administration with assurance of respect of confidentiality.

## Results

**Characteristics of study participants:** a total of 206 pregnant women, living in the New-Bell health district and ageing between 15 and 41 years (mean age=25.0; median age=25+/-5.5) were enrolled in the study. Table 1 presents socio-demographic characteristics of the study participants. From the table, it is noted that; 33.0% of pregnant women attending ANC at the New-Bell district hospital were aged between 20 to 24 years. Also, only 68.4% of them have gone to school. Up to 85.0% of the study participants were married.

**Table 1:** socio-demographic characteristics of participants

Indicators	Range /Option	Frequency(n)	Percentage
Age in years	15-19	32	15.5%
	20-24	68	33.0%
	25-29	67	32.5%
	30-34	30	14.6%
	35-39	6	2.9%
	40-44	3	1.5%
Marital status	Single	31	15.0%
	Married	175	85.0%
	Divorced	0	0.0%
	Widow	0	0.0%
Educational level	Never gone to school	65	31.6%
	Primary school	56	27.2%
	Secondary school	74	35.9%
	University	11	5.3%

### KAP on malaria

**Figure 1:**
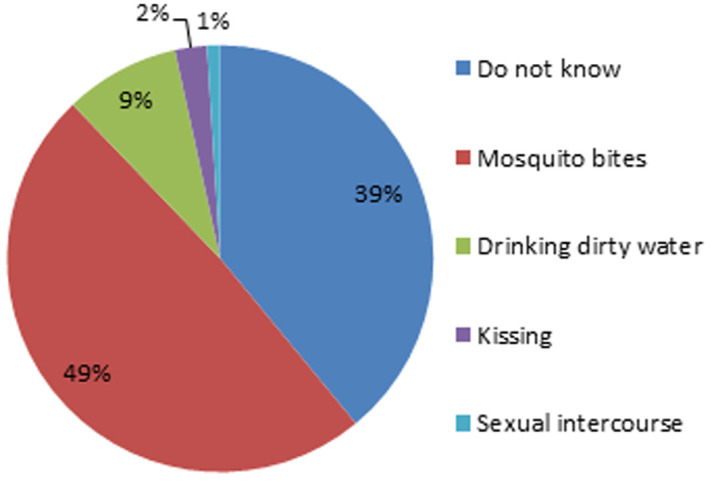
distribution of malaria transmission modes

**Knowledge on malaria:** all the 206 (100%) pregnant women surveyed had heard of malaria in the past. Figure 1 presents the main information channels from where pregnant women got aware of malaria. It is noted that, the main information channels were hospitals/health facilities and television (32.5% each), followed by community health worker (26.9%).

**Figure 2:**
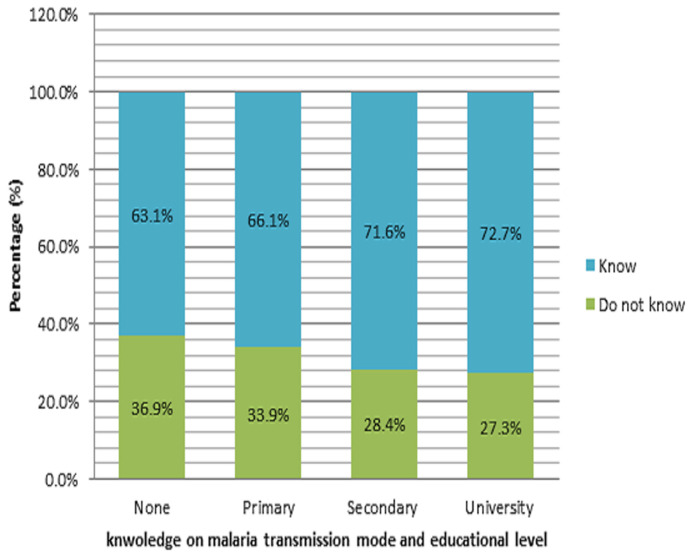
relationship between knowledge on malaria mode of transmission and educational level

**Knowledge on malaria etiology and mode of transmission:** concerning malaria transmission mode and etiology, over half (48.5%) of the pregnant women surveyed did not know that mosquito bites were the only cause and mode of transmission of malaria. Figure 1, shows the various transmissions mode presented by the women surveyed. It is noted that, up to 2.4% of them thought that kissing was the transmission mode. When stratifying by level of education, up to 36.9% of surveyed without knowledge on malaria etiology had never gone to school (Figure 2).

**Figure 3:**
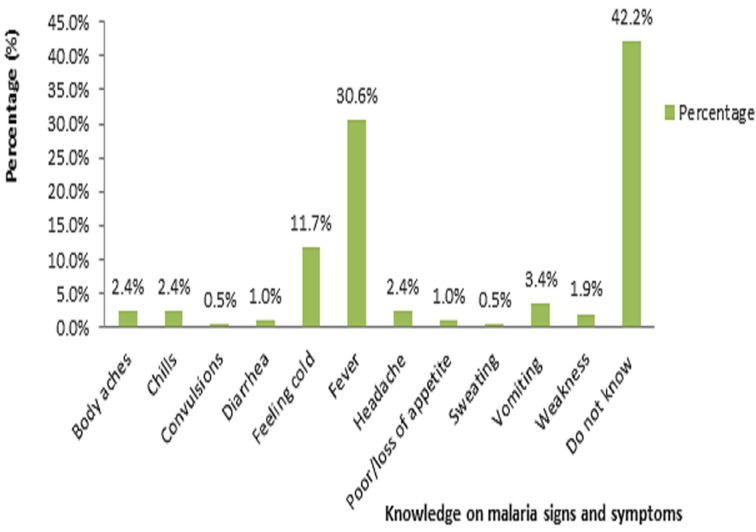
pregnant women´s knowledge on malaria signs and symptoms

**Figure 4:**
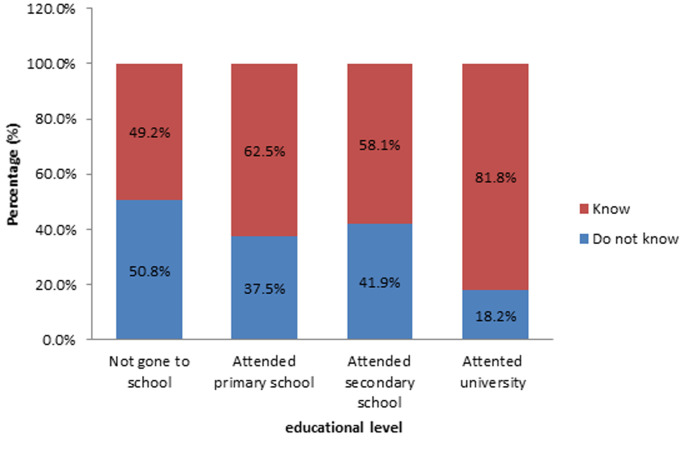
relationship between knowledge on malaria symptoms and educational level

**Knowledge on malaria signs and symptoms:** up to 42.2% of the study population did not know a symptom of malaria. Figure 3 presents women´s response regarding knowledge on malaria main sign and symptom. It is noted that 30.6% of them choose fever as the main malaria sign and symptom. Twenty four (11.7%) declared that feeling cold and 7 (6.9%) said vomiting were main malaria signs and symptoms. Up to 18.2% of participants who attended university did not know a malaria symptom. Figure 4presents women´s knowledge regarding malaria symptoms based on educational level. It is noted that most of the women without knowledge on malaria sign and symptom 50.8% had never gone to school.

**Figure 5:**
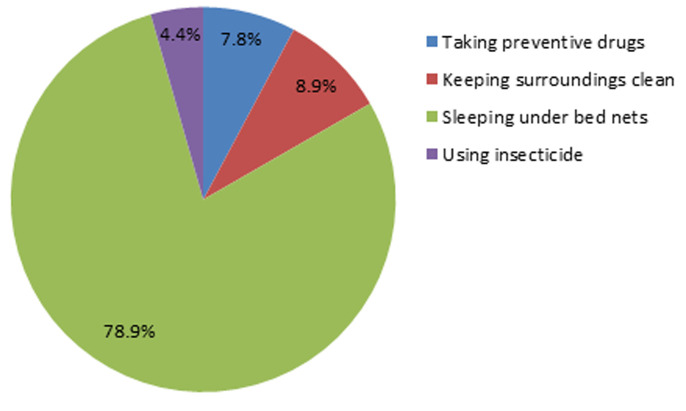
pregnant women´s knowledge on malaria prevention

**Figure 6:**
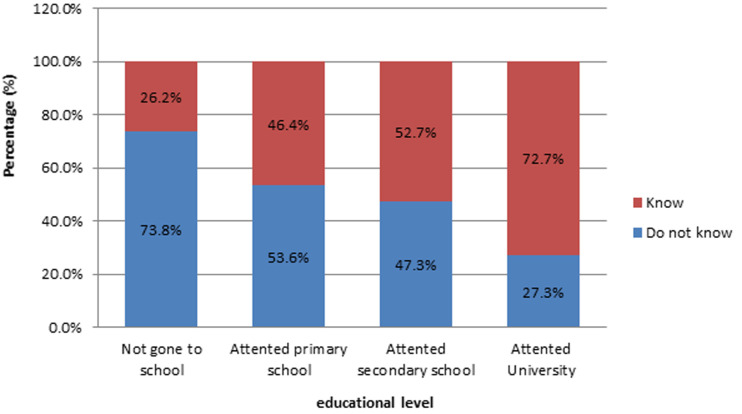
relationship between knowledge of surveyed on malaria preventive measures and educational level

**Knowledge on malaria preventive measures:** one hundred and sixteen (56.3%) of the pregnant women surveyed did not know a malaria preventive measure. Figure 5 presents the various malaria preventive measures stated by surveyed. Of the 43.7% who knew a preventive measure, only 78.9% knew that sleeping under mosquito nets prevents from contracting malaria. Up to 73.8% of the surveyed not knowledgeable on malaria preventive measures had never gone to school (Figure 6).

**Figure 7:**
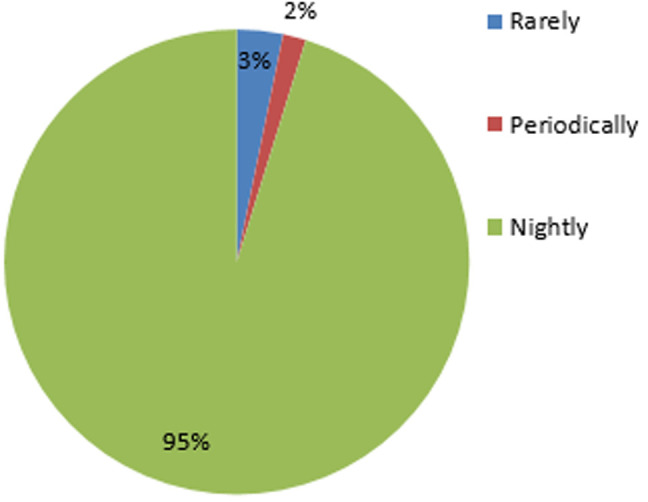
frequency of LLINs usage

**Attitudes and perceptions regarding malaria:** of the 206 pregnant women surveyed, only 60.2% (124/206) of them own and use a long lasting insecticide treated mosquito net. Of the 124 using LLINs, only 51.6% of them (64/124) treat the nets. Of the 124 using the nets, only 95.2% of them used the net nightly. Figure 7 presents the frequency at which the surveyed use LLINs.

## Discussion

This study aimed at exploring pregnant women´s knowledge, attitudes and perceptions regarding malaria. Women surveyed in the course of this study were attending ANC in the New-Bell district hospital located in the Douala II municipality of Cameroon.

**Pregnant women´s knowledge on malaria:** the study revealed that, all the surveyed had heard of malaria in the past as in a study done by Bamaga *et al*. in Hadhramout [15]. This is not a novel situation and is online with the findings of Hanafi-Bodj *et al*. [16]. Such findings are explained by the fact that, all the surveyed live villages of the endemic swampy littoral region of Cameroon were inhabitants are frequently exposed to mosquito bites and where malaria transmission occurs throughout the year [17]. Informations on malaria are mostly heard from health facilities and television channels. This shows that health workers represent one of the most important dissemination channels regarding malaria. This finding is in line with the reports of Kimbi *et al*. following a survey of women living in the Buea Health District. Also, television channels stand good chances in disseminating informations on malaria but, there is a need to focus on malaria causative agent, signs and symptoms as well as malaria preventive measures. This can be done by organizing community sensitization day on malaria, community group discussion on malaria [18,19]. Although all the study participants heard of malaria in the past, they have poor knowledge on malaria etiology with only 48.5% of them knowing that mosquito bites are the only mode of malaria transmission.

This finding differs from what was discovered by Kimbi *et al*. who recorded 86.2% while Nsagha *et al*.in such a study done in the Ndu HD and recorded 27.9% correct answer on malaria mode of transmission [20]. This finding was worst among illiterate participants with up to 39.6% of them uninformed on malaria mode of transmission. Such findings were also shown by Mazigo *et al*. in a study carried out in rural Northwest of Tanzania [17]. This poor knowledge on malaria etiology and mode of transmission can hinder the activities of surveillance and control of malaria in malaria endemic countries such as Cameroon. This poor knowledge on malaria mode of transmission can be attributed to the fact that most of the study participant had never gone to school and had lots of misconceptions on malaria etiology and transmission. This observation was recorded in studies done in Ethiopia and Nigeria [13,18]. Findings of this study revealed poor knowledge on malaria etiology, transmission and prevention with the situation being worst amongst illiterate participants. This can have an impact on malaria control when planning sustainable and efficient malaria control strategies.

**Pregnant women´s attitudes and perceptions regarding malaria:** knowing that sleeping under mosquito nets is one of the main practice in vector control, only 60.2% of study participants own a mosquito nets amongst whom 1.6% used it periodically (only during rainy season). Mosquito net usage drops among owner during warming season there is a need to improve sensitization on the foremost importance of mosquito nets no matter the season.

**Implication to policy:** improvement in education on malaria during ANC; implication of women in malaria control programs; promotion of frequent talk show and group discussion on malaria to reduce risk of exposure and transmission.

**Future research:** investigation of malaria prevalence among pregnant women attending ANC in the New-Bell district hospital; exploration KAP regarding HIV/AIDS in pregnant women attending ANC in the New-Bell district hospital; investigation of HIV/AIDS prevalence in pregnant women attending ANC in the New-bell district hospital.

## Conclusion

Malaria remains a major public health problem in Cameroon. There is a need to improve education on malaria at community base with the implication of women in talk shows, group discussion and malaria control campaigns.

### What is known about this topic

- Pregnant women are knowledgeable on malaria;
- Pregnant women perceived the importance of LLINs;
- Information on malaria is mostly disseminated by health personnels.

### What this study adds

- Pregnant women of this study area had poor knowledge regarding malaria and do not make use of LLINs; there is a need to implicate them in malaria control programs and discussions;
- Most of the surveyed had never gone to school. This situation might also be the cause of poor knowledge on malaria and might expose them to several pathologies. There is a need to promote education in the study area;
- Television was also presented as a main dissemination channel. Implementation of malaria control measures can be done through dissemination of key messages in television channels.
